# Supplementary material for: The neural crest is a source of mesenchymal stem cells with specialized hematopoietic stem cell niche function
Source: eLife. 2014 Sep 25;3:e03696. doi: 10.7554/eLife.03696 (PMC4381911; doi:10.7554/eLife.03696)
Supplement: Supplementary file 1. — Summary of mouse strains used in this study. DOI: http://dx.doi.org/10.7554/eLife.03696.019 [file elife03696s001.docx]

**MOUSE STRAINS**

**SHORT NAME TRANSGENE SYMBOL I.D. ORIGINAL NAME REF. DESCRIPTION**

*Nes-Gfp* *Tg(Nes-EGFP)33^Enik^* MGI:*5523870 Nestin-GFP* [1] EGFP expression driven by regulatory elements and neural enhancers

from rat *nestin* gene.

*R26-iDTA* *Gt(ROSA)26Sor^tm2(DTA)Riet^* MGI:*3653504* *R26:lacZbpA^flox^DTA* [2] *Allele* for tamoxifen-inducible diphtheria toxin (DT) expression.

*R26-iDTR* *Gt(ROSA)26Sor^tm1(HBEGF)Awai^* MGI:*3772576* *iDTR*  [3] *Allele* for tamoxifen-inducible diphtheria toxin receptor (DTR) expression.

***Lineage specific Cre-driver alleles***

*Nes-CreER^T2^ Tg(Nes-cre/ERT2)1^Fsh^* MGI:*3774417* *Nestin^CreERT2^* [4] Tamoxifen-induclible *cre* recombianse under control of rat *nestin*

regulatory elements.

*Col2.3-Cre Tg(Col1a1-cre)1^Kry^* MGI:*3041864 2.3-kb α1(I)-collagen-Cre* [5] *Cre* recombinase allele driven by *collagen type I* gene regulatory

elements. Expression mostly restricted to maturing osteoblastic cells.

*Hoxb6-CreER* *Tg(Hoxb6-cre/ERT)1^Smac^* MGI:*3793474* *Hoxb6CreER^T^* [6] Tamoxifen-inducible *Cre* recombinase driven by *Hoxb6* regulatory

elements. Expression mostly restricted to lateral mesoderm.

*Wnt1-Cre2 Tg(Wnt1-cre)2^Sor^* MGI:*5485027* *Wnt1-Cre2* [7] Mouse *Wnt1* promoter-driven *Cre* recombinase *allele.* Expressed in

neural crest derived lineages.

*Sox10-CreER* *Tg(Sox10-cre/ERT2)26^Vpa^* MGI:*5301107* *Sox10::iCreER^T2^* [8] Tamoxifen-inducible *Cre* recombinase allele driven by *Sox10* regulatory

region. Restricted to Neural crest and Schwann cell precursor-derived

lineages.

*Dhh-Cre* *Tg(Dhh-cre)1^Mejr^* MGI:*4359600* *Dhh-cre*  [9] Desert hedgehog (*Dhh*) promoter-driven *Cre* recombinase

*allele*. Expression restricted to glial lineages.

***Cre-reporter alleles***

*KFP* *Tg(CAG-LSL-KFP)* [10] Far red KFP fluorescent protein constitutively expressed upon

*Cre*-mediated recombination.

*RCE* *Gt(ROSA)26Sor^tm1.1(CAG-EGFP)Fsh^* MGI:*4412373* *RCE:loxP* [11] EGFP fluorescent protein expressed upon *Cre*-mediated

recombination from *Rosa26* locus.

*R26-Tomato Gt(ROSA)26Sor^tm14(CAG-tdTomato)Hze^* MGI:*3809524* *Ai14* [12] tdTomato fluorescent protein expressed upon *Cre*-mediated

recombination from *Rosa26* locus.

***Null and conditional alelles***

*Erbb3 ko* *Erbb3^tm2Cbm^* MGI:*1929598* *erbB3***^Δ^** [13] *Null* allele for mouse *Erbb3* gene.

*Erbb3-floxed* *Erbb3^tm3Cbm^* MGI:*3835516* *ErbB3^flox^* [14] *LoxP*-flanked conditional *allele* for mouse *Erbb3* gene.

*Cxcl12-floxed* *Cxcl12^tm1.1Ystz^*  MGI:*4888352* *Cxcl12^F^* [15] *LoxP*-flanked conditional *allele* for mouse *Cxcl12* gene.

**References**

[1] Mignone JL *et al.* 2004

[2] Brockschnieder D *et al.* 2006

[3] Buch T *et al.* 2005

[4] Balordi F *et al.* 2007

[5] Dacquin R *et al.* 2002

[6] Nguyen MT *et al.* 2009

[7] Lewis AE *et al.* 2013

[8] Laranjeira C *et al*. 2011

[9] Jaegle M *et al.* 2003

[10] Diéguez-Hurtado R *et al.* 2010

[11] Sousa VT *et al.* 2009

[12] Madisen L *et al.* 2010

[13] Riethmacher D *et al.* 1997

[14] Brinkmann B *et al.* 2008

[15] Tzeng YS *et al.* 2011
